# Ultrastructural, Antioxidant, and Metabolic Responses of Male Genetically Improved Farmed Tilapia (GIFT, *Oreochromis niloticus*) to Acute Hypoxia Stress

**DOI:** 10.3390/antiox13010089

**Published:** 2024-01-10

**Authors:** Yifan Tao, Jixiang Hua, Siqi Lu, Qingchun Wang, Yan Li, Bingjie Jiang, Yalun Dong, Jun Qiang, Pao Xu

**Affiliations:** 1 Key Laboratory of Freshwater Fisheries and Germplasm Resources Utilization, Ministry of Agriculture and Rural Affairs, Freshwater Fisheries Research Center, Chinese Academy of Fishery Sciences, Wuxi 214081, Chinajiangbingjie@ffrc.cn (B.J.);; 2 Wuxi Fisheries College, Nanjing Agricultural University, Wuxi 214081, China

**Keywords:** hypoxia, GIFT, oxidative stress, metabolic adaptation, histology

## Abstract

Tilapia tolerate hypoxia; thus, they are an excellent model for the study of hypoxic adaptation. In this study, we determined the effect of acute hypoxia stress on the antioxidant capacity, metabolism, and gill/liver ultrastructure of male genetically improved farmed tilapia (GIFT, *Oreochromis niloticus*). Fish were kept under control (dissolved oxygen (DO): 6.5 mg/L) or hypoxic (DO: 1.0 mg/L) conditions for 72 h. After 2 h of hypoxia stress, antioxidant enzyme activities in the heart and gills decreased, while the malondialdehyde (MDA) content increased. In contrast, in the liver, antioxidant enzyme activities increased, and the MDA content decreased. From 4 to 24 h of hypoxia stress, the antioxidant enzyme activity increased in the heart but not in the liver and gills. Cytochrome oxidase activity was increased in the heart after 4 to 8 h of hypoxia stress, while that in the gills decreased during the later stages of hypoxia stress. Hypoxia stress resulted in increased Na^+^-K^+^-ATP activity in the heart, as well as hepatic vacuolization and gill lamella elongation. Under hypoxic conditions, male GIFT exhibit dynamic and complementary regulation of antioxidant systems and metabolism in the liver, gills, and heart, with coordinated responses to mitigate hypoxia-induced damage.

## 1. Introduction

The dissolved oxygen (DO) level in water is inherently unstable and is affected by water quality, weather, temperature, and cultivation density [1]. As a result of this unpredictability, fish are frequently exposed to suboptimal DO conditions, which trigger a hypoxia response that leads to detrimental effects on their behavior, physiology, growth, and survival [2]. With the development of aquaculture, the prevalence of high-density cultivation and the exacerbation of environmental stressors have made low-DO stress an increasingly frequent occurrence.

Fish under acute hypoxia stress exhibit various morphological changes that can be detected by histological analyses. As the primary respiratory system component in fish, gills play a pivotal role in various essential physiological processes, and they are highly sensitive to variations in DO [3]. Under acute hypoxia stress, Crucian carp (*Carassius carassius*) undergo gill remodeling via interlamellar cell mass (ILCM) regression, leading to an increase in gill surface area [4]. Other studies have shown that the gill lamellae are elongated in Lake Qinghai scaleless carp (*Gymnocypris przewalskii*) [5] and mangrove killifish (*Kryptolebias marmoratus*) [3] under acute hypoxia stress. These findings imply that the oxygen uptake capacity of fish can be enhanced under low-DO conditions via gill remodeling. The liver is an essential organ in fish that aids in their adaptation to fluctuating environmental conditions. Studies of large yellow croaker (*Larimichthys crocea*) [6] and common carp (*Cyprinus carpio*) [7] have demonstrated significant pathological changes, including hepatocyte swelling and cytoplasmic vacuolization, in the liver tissues of these fishes under acute hypoxia stress. However, little is known about the structural changes in the gill and liver tissues of genetically improved farmed tilapia (GIFT, *Oreochromis niloticus*) under acute hypoxia stress.

In fish under acute hypoxia stress, endogenous antioxidant defense mechanisms play a crucial role in regulating reactive oxygen species (ROS) production and in minimizing oxidative damage [8]. The antioxidant enzyme system, which involves superoxide dismutase (SOD), catalase (CAT), and glutathione peroxidase (GSH-Px), serves as the first line of defense against ROS [9]. Generally, the activity of these antioxidant enzymes is adjusted to counter the oxidative stress induced by hypoxia [10]. For example, there is a significant rise in the activity of antioxidant enzymes in the heart, liver, and gills of Gobbid fish (*Odontobutis potamophila*) under hypoxia stress [11], and activation of the endogenous antioxidant defense system partly alleviates the oxidative stress damage caused by hypoxia in common carp [12].

Hypoxia stress also disrupts an organism’s physiological and metabolic balance. Fish responses to hypoxia stress involve complex physiological and biochemical processes, including a decrease in the metabolic rate and an increase in anaerobic metabolism [13]. When the DO level is insufficient to meet the aerobic demands of glycolysis, fish are unable to maintain their normal physiological functions, and anaerobic metabolism is passively enhanced to provide energy [14,15]. Research shows that aquatic organisms can adapt to low-DO environments by adjusting the activity of metabolism-related enzymes to modify energy supply pathways [16]. Na^+^-K^+^-ATPase is a key enzyme for Na^+^/K^+^ pump activity, the primary functions of which are to maintain ionic permeability of the cytoplasmic membrane and to participate in essential biochemical processes such as cellular energy metabolism, substance transport, and oxidative phosphorylation [17]. Lactate dehydrogenase (LDH) and cytochrome oxidase (COO) are two crucial regulatory enzymes in energy metabolism [18,19]. To some extent, the activities of Na^+^-K^+^-ATPase, LDH, and COO reflect the effect of hypoxia stress on fish metabolism.

GIFT bred from selected tilapia breeding stocks from Africa and Asia have gained prominence in China in recent years because of their improved growth, high yield, and strong adaptability [20]. Based on our prior research [21] and preliminary investigations conducted for this study, we determined that GIFT can endure a low-DO environment (DO = 1.0 mg/L) for 72 h without experiencing mortality, indicating that they are an ideal candidate for investigating the mechanisms underlying hypoxia response. Recent studies have reported that acute hypoxia stress induces a glycolipid metabolism response in the blood [22] and liver [21] of GIFT. Furthermore, the regulatory mechanisms of two non-coding RNAs, namely microRNA (miR)-34a [23] and miR-92a [24], were investigated in GIFT in response to hypoxia stress. However, a detailed understanding of the antioxidant and metabolic response mechanisms of GIFT to hypoxic stress remains to be elucidated.

Tilapia show pronounced sexual dimorphism, with males outpacing the growth of females by 30% [25]. In China, the promotion of a high-male or monosex male tilapia culture has proven effective in enhancing the yield of marketable fish, thereby optimizing farming efficiency. Therefore, the objectives of this study were to investigate the antioxidant capacity, metabolic responses, and morphological changes in the gill, liver, and heart tissues of male GIFT under acute hypoxic conditions. With this study, we aim to enhance our understanding of the dynamic tissue response in fish exposed to acute hypoxia stress.

## 2. Materials and Methods

### 2.1. Animals

GIFT larvae were bred in house at the Yangzhong Base of the Freshwater Fisheries Research Center (FFRC) of the Chinese Academy of Fishery Sciences for three months. Following this period, males and females were separated upon the appearance of external genitalia [26]. Healthy and vigorous male GIFT (60–80 g) were chosen as the experimental fish. Prior to the experiment, the fish underwent a 15-day acclimation period in 600 L indoor plastic tanks equipped with a recirculating system, where the water temperature was maintained at 28–29 °C, the pH level was 7.4–7.6, the DO level was 6.5–7.0 mg/L, the water alkalinity level was 0.8–1.0 mmol/L, and the water hardness was 95–100 mg/L, with a 12 h light/12 h dark photoperiod. During the acclimation period, the fish were fed commercial feed (29% crude protein, 6% crude fat) equaling 5% of their body weight twice daily at 8:00 and 16:00. One-third of the water was changed every three days. The DO level was determined using a DO meter (LD0101, Hach, Loveland, CO, USA). The temperature, pH, and hardness level were measured using a multiparameter water quality analyzer (SL1000, Hach, Loveland, CO, USA). The alkalinity was assessed via acidimetric titrations [27].

### 2.2. Acute Hypoxia Treatment

A total of 240 male GIFT fish, averaging 13.4 ± 0.112 cm in body length and 75.7 ± 1.393 g in body weight, were utilized for acute hypoxia stress experiments. These fish were randomly distributed across six tanks, each with dimensions of 1200 mm (diameter) × 1000 mm (height), accommodating 40 fish per tank. Three tanks were used for hypoxic stress treatment (DO: 1.0 ± 0.1 mg/L; hypoxia group, HG), while the other three tanks were maintained under normal DO conditions (DO: 6.5 ± 0.1 mg/L; control group, CG). To rapidly achieve the target DO concentration in the three tanks (the hypoxia group), a plastic film was used to cover the tank, and nitrogen gas was introduced at a flow rate of 25 mL/s for 25–30 min [28]. DO levels were monitored hourly throughout the experimental period, and the DO level was controlled by adjusting the flow rates of air into the water [21]. Other environmental conditions were the same as those during the acclimation phase. The hypoxia treatment lasted for 72 h.

### 2.3. Sampling

At various time points during the hypoxia treatment or non-treatment (control conditions) (0, 2, 4, 8, 24, 48, and 72 h), three fish were randomly selected from each tank. Following anesthesia using 100 mg/L MS-222, the gill, liver, and heart tissues of the GIFT were collected and immediately frozen in liquid nitrogen. These samples were stored at −80 °C until further processing. Our prior research [29] indicated that, in the short term (96 h) and under non-stress conditions, there are no observable changes in GIFT liver and gill tissues. Therefore, only two fish were sampled from each hypoxia treatment tank at 0, 8, 24, and 72 h. Subsequently, these fish were dissected to obtain the second branchial arches from the gill and liver tissues for histological analyses. Following the experiment, the surviving experimental fish were temporarily retained in the tank for a week before being reintroduced into the pond.

### 2.4. Histological Analyses

The liver and gill tissue samples were fixed in Bouin’s solution for 24 h, then rinsed several times with phosphate-buffered saline (PBS). Gill tissue decalcification was carried out using decalcification fluid (YCOO, Shanghai, China) at a temperature of 27 °C. Livers and softened gills were dehydrated in a graded alcohol series, cleared in xylene, embedded in paraffin, and cut into 5 μm sections. These sections were stained with hematoxylin and eosin, sealed with neutral resin, and observed under a DFC495 light microscope (Leica Microsystems, Wetzlar, Germany) to detect structural changes. Furthermore, the hepatic vacuole area, gill lamellae length, and ILCM thickness were measured using Image-Pro plus v6.0.0.260 software (Media Cybernetics, Bethesda, MD, USA). Tissue sections from GIFT exposed to 0, 8, 24, and 72 h of hypoxic stress treatment were analyzed (six images per sampling time). The hepatic vacuole area was quantified following the methods recommended by Horai et al. [30] and expressed as relative vacuole area ((%) = (vacuole area/total liver area) × 100). For each image, three secondary lamellae were randomly selected, and the length of the gill lamellae beyond the ILCM was measured. Additionally, the ILCM thickness was measured at three randomly chosen locations within each image.

### 2.5. Measurement of Antioxidant and Metabolic Parameters

After snap freezing in liquid nitrogen, each sample (approximately 0.1 g) was placed in a sterile 1.5 mL tube; then, prechilled PBS was added at a ratio of 1:9 *w*/*v*. The mixture was then centrifuged (4 °C, 3000× *g*) for 20 min, and the supernatant was collected for determination of enzyme activity and malondialdehyde (MDA) content analyses. The MDA concentration and the SOD, CAT, GSH-Px, glutathione reductase (GR), glutathione S-transferase (GST), Na^+^-K^+^-ATPase, LDH, and COO activities were determined using commercial kits (Nanjing Jiancheng Bioengineering Institute, Nanjing, China).

SOD activity was assessed by measuring the inhibitory effect of superoxide radicals on the cytochrome reduction rate (c) at 450 nm [31]. CAT activity was assayed by detecting the residual H_2_O_2_ absorbance at 405 nm [32]. The MDA content was determined based on the condensation of MDA with thibabital acid, which forms a red product, utilizing an absorbance method with a maximum absorption peak at 532 nm [33]. GSH-Px activity was evaluated by examining the NADPH oxidation rate [34]. GR’s catalytic effect involves supplying hydrogen through NADPH to reduce GSSG to GSH. GR activity was determined by detecting the decrease in the NADPH absorbance value [35]. GST catalyzes the reduction of GSH upon binding to the 1-chloro-2,4-dinitrobenzene substrate. GSH activity was determined by measuring the decrease in GSH concentration per minute via absorbance measurements [36]. 

LDH catalyzes the interconversion of pyruvate and lactate. The reaction between pyruvate and dinitrophenylhydrazine produces pyruvate dinitrophenylhydrazone, which is brownish–red in color, with a maximum absorption peak at 450 nm [37]. COO activity was assessed by the catalysis of mitochondrial complex IV, generating oxidized cytochrome C from reduced cytochrome C, using an absorbance method with a maximum absorption peak at 550 nm [38]. Na^+^-K^+^-ATPase activity was determined following the methodology outlined by McCormick [39].

### 2.6. Statistical Analyses

All data are presented as means ± standard error (means ± SE). Data were analyzed using SPSS 22.0 software (SPSS Inc., Chicago, USA). The normality and homogeneity of variance were assessed using the Shapiro–Wilk and Levene tests, respectively. A repeated-measures ANOVA was used for statistical analyses [40,41]. Multiple comparisons were performed if there was a main effect. A simple effect analysis was performed if there was an interaction effect between time and the DO level. Differences were considered significant at *p* < 0.05.

## 3. Results

### 3.1. Effect of Acute Hypoxic Stress on Antioxidant and Metabolic Regulation in the Heart

**Figure 1:**
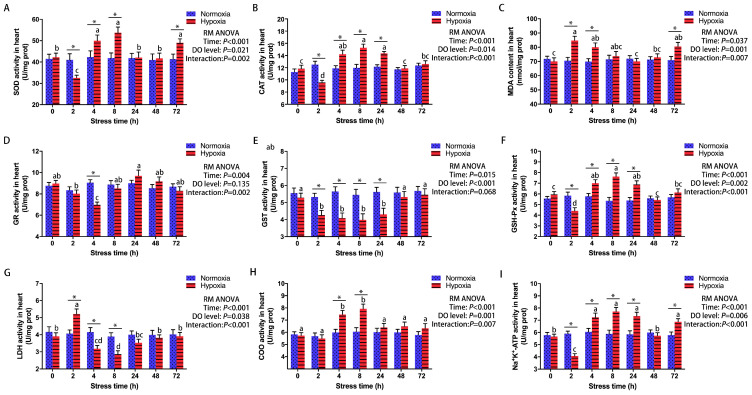
Effect of 72 h of hypoxia stress on antioxidant and metabolic indexes in the hearts of male genetically improved farmed tilapia (GIFT) (*n* = 9). (**A**) Superoxide dismutase, SOD; (**B**) catalase, CAT; (**C**) malondialdehyde, MDA; (**D**) glutathione reductase, GR; (**E**) glutathione S-transferase, GST; (**F**) glutathione peroxidase, GSH-Px; (**G**) lactate dehydrogenase, LDH; (**H**) cytochrome oxidase, COO; (**I**) Na^+^-K^+^-ATPase. Asterisks (*) indicate statistically significant differences (*p* < 0.05) between hypoxia-treated fish and the and control groups. Different letters indicate statistically significant differences (*p* < 0.05) among different time points within the same treatment group. In the figure, “prot” is the abbreviation of “protein”, “RM ANOVA” is the abbreviation of “repeated-measures ANOVA”, and “DO” is the abbreviation of “dissolved oxygen”.

Significant changes in antioxidant and metabolic indexes in the hearts of male GIFT after 72 h of hypoxic stress (DO 1.0 mg/L) were identified via a repeated-measures ANOVA (Figure 1). Compared to the CG, heart SOD, CAT, and Na^+^-K^+^-ATPase activities (Figure 1A,B,I) in the HG exhibited a decrease at 2 h (SOD, *p* = 0.021; CAT, *p* < 0.001; Na^+^-K^+^-ATPase, *p* < 0.001), followed by an increase at 4 h (SOD, *p* = 0.034; CAT, *p* = 0.009; Na^+^-K^+^-ATPase, *p* = 0.016). After 72 h, heart SOD and Na^+^-K^+^-ATPase activities remained significantly higher (SOD, *p* = 0.021; Na^+^-K^+^-ATPase, *p* = 0.007) in the HG than in the CG, while heart CAT activity in the HG decreased to the same level as that in the CG. At 2, 4, and 72 h, heart MDA contents (Figure 1C) were significantly higher (2 h, *p* = 0.002; 4 h, *p* = 0.012; 72 h, *p* = 0.022) in the HG than in the CG. At 4 h, heart GR activity (Figure 1D) was significantly lower (*p* < 0.001) in the HG than in the CG. Compared with the CG, heart GST activity (Figure 1E) in the HG decreased at 2 h (*p* = 0.008) but returned to the normal level by 48 h. Furthermore, heart GSH-Px activity (Figure 1F) in the HG decreased at 2 h (*p* = 0.004), then increased at 4 h (*p* = 0.008) but dropped to control levels after 48 h. Notably, the variations in heart LDH activity (Figure 1G) were the opposite to those observed in heart GSH-Px activity under hypoxia stress. The heart COO activity (Figure 1H) in the HG increased with prolonged hypoxia stress, peaking at 8 h (*p* = 0.002).

### 3.2. Effect of Acute Hypoxic Stress on Antioxidant and Metabolic Regulation in the Liver

**Figure 2:**
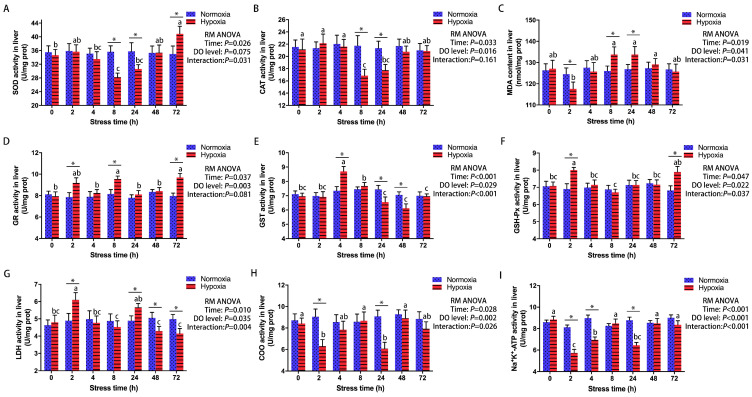
Effect of 72 h of hypoxia stress on antioxidant and metabolic indexes in the livers of male GIFT (*n* = 9). (**A**) Superoxide dismutase, SOD; (**B**) catalase, CAT; (**C**) malondialdehyde, MDA; (**D**) glutathione reductase, GR; (**E**) glutathione S-transferase, GST; (**F**) glutathione peroxidase, GSH-Px; (**G**) lactate dehydrogenase, LDH; (**H**) cytochrome oxidase, COO; (**I**) Na^+^-K^+^-ATPase. Asterisks (*) indicate statistically significant differences (*p* < 0.05) between hypoxia-treated groups and control groups. Different letters indicate statistically significant differences (*p* < 0.05) among different time points within the same treatment group. In the figure, “prot” is the abbreviation of “protein”, “RM ANOVA” is the abbreviation of “repeated-measures ANOVA”, and “DO” is the abbreviation of “dissolved oxygen”.

Repeated-measures ANOVAs suggested that antioxidant and metabolic responses in the livers of male GIFT were significantly influenced by the sampling time and the DO level (Figure 2). In comparison to the CG, hepatic SOD and CAT activities (Figure 2A,B) in the HG decreased at 8 h (SOD, *p* = 0.003; CAT, *p* = 0.026) and gradually increased as the hypoxia stress treatment progressed. By 72 h, the hepatic SOD activity was significantly higher (*p* = 0.034) in the HG than in the CG. The MDA content (Figure 2C) in the liver was significantly lower in the HG than in the CG at 2 h (*p* = 0.028) but significantly higher in the HG than in the CG at 8 and 24 h (8 h, *p* = 0.017; 24 h, *p* = 0.039). Hepatic GR activity (Figure 2D) was significantly higher in the HG than in the CG at 2, 8, and 72 h (2 h, *p* = 0.018; 8 h, *p* = 0.013; 72 h, *p* = 0.002). Compared to the CG, hepatic GST activity (Figure 2E) in the HG increased at 4 h (*p* = 0.008) and decreased at 24 h (*p* = 0.033). However, by 72 h, this activity had dropped to the control level. At 2 and 72 h, the hepatic GSH-Px activity was significantly higher (2 h, *p* = 0.006; 72 h, *p* = 0.022) in the HG than in the CG (Figure 2F). The hepatic LDH activity (Figure 2G) was significantly higher in the HG than in the CG at 2 and 24 h (2 h, *p* = 0.028; 24 h, *p* = 0.031) but significantly decreased in the HG at 48 and 72 h (48 h, *p* = 0.033; 72 h, *p* = 0.024). Compared to the CG, hepatic COO and Na^+^-K^+^-ATPase activities (Figure 2H,I) in the HG decreased at 2 h (COO, *p* = 0.01; Na^+^-K^+^-ATPase, *p* < 0.001) but returned to normal levels after 48 h.

### 3.3. Effect of Acute Hypoxic Stress on Antioxidant and Metabolic Regulation in Gills

**Figure 3:**
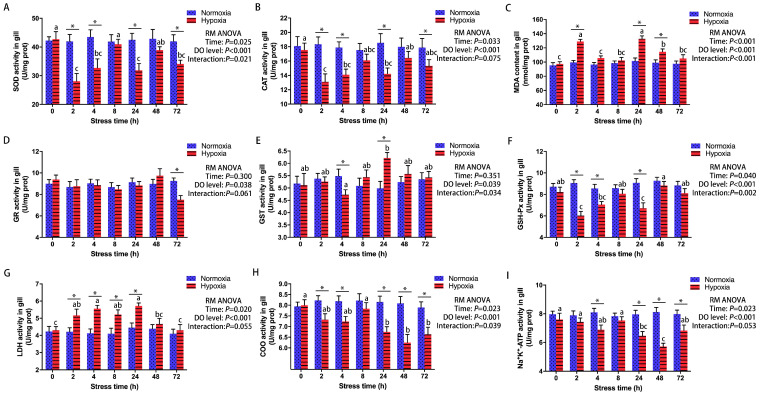
Effect of 72 h of hypoxia stress on antioxidant and metabolic indexes in the gills of male GIFT (*n* = 9). (**A**) Superoxide dismutase, SOD; (**B**) catalase, CAT; (**C**) malondialdehyde, MDA; (**D**) glutathione reductase, GR; (**E**) glutathione S-transferase, GST; (**F**) glutathione peroxidase, GSH-Px; (**G**) lactate dehydrogenase, LDH; (**H**) cytochrome oxidase, COO; (**I**) Na^+^-K^+^-ATPase. Asterisks (*) indicate statistically significant differences (*p* < 0.05) between hypoxia-treated groups and control groups. Different letters indicate statistically significant differences (*p* < 0.05) among different time points within the same treatment group. In the figure, “prot” is the abbreviation of “protein”, “RM ANOVA” is the abbreviation of “repeated measures ANOVA”, and “DO” is the abbreviation of “dissolved oxygen”.

The effects of 72 h of hypoxia stress on antioxidant and metabolic parameters in the gills are shown in Figure 3. At all sampling times, except 8 and 48 h, the SOD and CAT activities (Figure 3A,B) in the gills were significantly lower in the HG than in the CG. In contrast, gill MDA contents (Figure 3C) were significantly higher in the HG than in the CG at 2, 24, and 48 h (2 h, *p* < 0.001; 24 h, *p* < 0.001; 48 h, *p* = 0.014). After 72 h of hypoxia stress, the gill GR activity was significantly decreased (*p* = 0.002) (Figure 3D). The activities of GST (Figure 3E) and GSH-Px (Figure 3F) in the gills were significantly lower (GST, *p* = 0.041; GSH-Px, *p* = 0.006) in the HG than in the CG at 4 h. However, at 24 h, the gill GST activity was significantly higher (*p* = 0.003) in the HG than in the CG, while the gill GSH-Px activity was still significantly lower (*p* = 0.002) in the HG than in the CG. Gill COO and Na^+^-K^+^-ATPase activities exhibited patterns similar to those of gill GSH-Px activity under hypoxia stress (Figure 3H,I). In comparison to the CG, the HG exhibited elevated gill LDH activity, with the highest values observed after the 4 and 24 h hypoxia treatments (4 h, *p* < 0.001; 24 h, *p* = 0.001) (Figure 3G). 

### 3.4. Effects of Acute Hypoxia Stress on Liver Histological Structure

**Figure 4:**
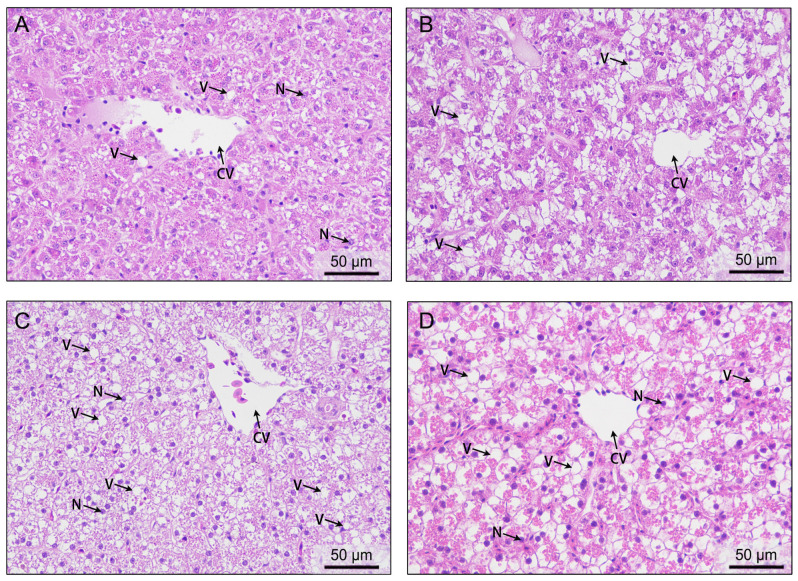
Effects of 72 h of hypoxia stress on liver histological structure. (**A**) Hypoxia at 0 h; (**B**) hypoxia at 8 h; (**C**) hypoxia at 24 h; (**D**) hypoxia at 72 h. Magnification: 400×. N: nuclei; V: vacuoles; CV: central veins.

Before hypoxic stress, the structural integrity and internal cell morphology in liver tissues were normal, with clear boundaries between cells (Figure 4A). After 8 and 24 h of hypoxia stress, a large number of liver cells had become vacuolated, with the nuclei located at the edge of the cells (Figure 4B,C). The relative vacuole area was significantly larger (*p* < 0.001) after 8 and 24 h of hypoxia stress than at 0 h (Table 1). The relative vacuole area had decreased after 72 h of hypoxic stress, but it was still significantly larger than that at 0 h (Figure 4D).

### 3.5. Effects of Acute Hypoxia Stress on Gill Histological Structure

**Figure 5:**
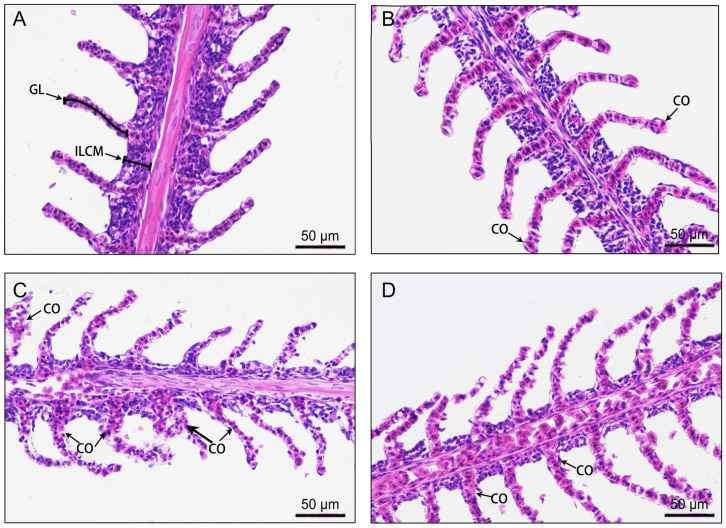
Effects of 72 h of hypoxia stress on gill histological structure. (**A**) Hypoxia at 0 h; (**B**) hypoxia at 8 h; (**C**) hypoxia at 24 h; (**D**) hypoxia at 72 h. Magnification: 400×. The measurement method for the length of gill lamellae and interlamellar cell mass thickness is shown in (**A**). CO: congestion; GL: gill lamellae; ILCM: interlamellar cell mass.

Before hypoxic stress, the gill filaments and lamellae had intact and well-organized structures (Figure 5A). However, after 8 h of hypoxia stress, signs of lamellae congestion were observed (Figure 5B). Lamellae congestion became more severe at 24 h (Figure 5C) but ameliorated after 72 h (Figure 5D). Utilizing Image-Pro plus software, we found that the length of the gill lamellae significantly increased (*p* < 0.001), while the ILCM thickness significantly decreased (*p* < 0.001) during the hypoxia stress treatment (Table 2). These results show that the gills underwent adaptive changes under acute hypoxia stress.

## 4. Discussion

The DO level is a key limiting factor in farmed fish production, as it is essential for their growth, reproduction, and metabolism [42,43]. Environmental factors, including water quality, weather variations, and temperature, can lead to a rapid decline (48–72 h) in the DO level in water [22,42,44,45]. GIFT demonstrate a robust tolerance to hypoxia, as evidenced by their short-term survival when DO levels decrease to 1.0 mg/L [21]. The mechanism underlying the hypoxic response in GIFT is still not fully understood. Our study suggests that under hypoxic conditions, male GIFT can dynamically and complementarily regulate the antioxidant systems and metabolism in the liver, gills, and heart, demonstrating a coordinated response to mitigate hypoxia-induced damage.

### 4.1. Tissue-Specific Responses to Oxidative Stress Induced by Hypoxia in Male GIFT 

A sudden reduction in DO levels in water induces ROS overproduction in aquatic organisms. The antioxidant enzymes SOD, CAT, and GSH-Px work together to convert superoxide radicals into oxygen and water, thus protecting the organism from oxidative damage [9]. In this study, male GIFT showed increased hepatic GSH-Px activities after 2 h of hypoxia stress, and this may have accelerated MDA decomposition and reduced its accumulation in the liver. However, both SOD and CAT activities in the liver were significantly decreased after 8 h of hypoxia stress, suggesting that acute hypoxia stress may affect the metabolic response in fish, reduce antioxidant enzyme activities, and exacerbate oxidative stress. The activities of SOD, CAT, and GSH-Px in the gills and heart were significantly decreased after 2 h of hypoxia stress, suggesting that the gills and heart were affected by oxidative stress earlier than the liver. The reason for this may be that the liver has a stronger antioxidant capacity and is therefore better able to maintain metabolism under oxidative stress. 

In fish, GST is present in various tissues, and it has antioxidant and detoxifying functions [46]. In this study, we found that hepatic GST activity was significantly increased after 4 h of hypoxia stress. The liver is an essential metabolic organ in fish, and GST activity upregulation might mitigate hepatic oxidative damage. The flavoenzyme GR catalyzes the conversion of oxidized glutathione into reduced glutathione, which reduces oxidative damage caused by free radicals [46]. In this study, the hepatic GR activity was significantly increased after 2, 8, and 72 h of hypoxia stress, while the GR activity in the gills and heart was somewhat inhibited. GR inhibition may affect the antioxidant function of the fish and lead to increased tissue damage. 

Different fish species exhibit tissue-specific antioxidant responses to hypoxia stress. SOD activities in the brain, liver, and gill tissues of common carp [12]; the CAT activity in the liver of goldfish (*Carassius auratus*) [47]; and GSH-Px activity in the liver and muscle of striped mullet (*Mugil cephalus*) [48] were all found to be increased under hypoxic conditions. In this study, the hearts, livers, and gills of male GIFT exhibited different antioxidant responses under hypoxia stress. The liver plays a crucial role in maintaining physiological functions in fish, and various metabolic processes in this organ are oxygen-dependent. Under hypoxia stress in male GIFT, liver protection might take precedence, i.e., the activities of its antioxidant enzymes (GR, GST, and GSH-Px) increase rapidly to reduce the effects of oxidative stress. The gills, on the other hand, may exhibit a weaker ability to cope with hypoxia stress. This is evidenced by the dynamic decrease in antioxidant enzyme activities (SOD, CAT, GR, and GSH-Px) in the gills of the HG compared to the CG throughout the hypoxia treatment, consistent with the results reported for hybrid yellow catfish (*Tachysurus fulvidraco* ♀ × *Pseudobagrus vachellii* ♂) [49]. As a vital organ in fish, the heart is responsible for powering blood flow to provide the body with oxygen and nutrients. Our results show that hypoxia-induced oxidative stress in the heart was alleviated within a short period (24–48 h) under the synergistic effect of antioxidant enzymes (SOD, CAT, and GSH-Px). However, after 72 h of hypoxia stress, the abnormal increase in MDA content in cardiac tissue suggests that male GIFT hearts are more sensitive to long-term oxygen deficiency than other tissues. This heightened sensitivity may exacerbate oxidative damage to the heart.

### 4.2. Shift in Energy Metabolism in Male GIFT under Hypoxia Stress 

Abrupt changes in water DO levels can influence the energy metabolism of fish [2]. Both LDH and COO are important enzymes in energy metabolism; LDH catalyzes the interconversion of pyruvate and lactate and serves as a marker for anaerobic metabolism in fish [50], whereas COO is the rate-limiting enzyme in the aerobic metabolism that catalyzes the reduction of oxygen molecules to water by transferring electrons generated during aerobic metabolism, thus producing ATP [51]. In this study, the LDH activity in the heart, liver, and gills significantly increased in the initial stages of hypoxia stress. Meanwhile, the COO activity in the gills and liver significantly decreased. Similar findings have been reported for hybrid yellow catfish [49]. These results suggest that short-term hypoxia stress may enhance the anaerobic metabolism but inhibit the aerobic metabolism in the gills and liver. However, we also detected significantly increased COO activity in the heart after 4 and 8 h of hypoxia stress, alongside significant decreases in heart LDH activity. The heart is responsible for pumping blood, delivering oxygen and various nutrients to tissues and organs, and removing metabolic end products. The increased COO activity in the heart may contribute to enhanced blood flow and oxygen utilization, ultimately improving adaptability to hypoxia stress. Hypoxia stress may stimulate aerobic metabolism in the heart in the short term, thereby alleviating the metabolic burden.

Hypoxia stress can also affect other metabolic pathways in fish, and studying these pathways can help us to understand the regulatory mechanisms involved in the hypoxia stress response. During the embryonic development of Japanese medaka (*Oryzias latipes*), embryo ATP and phosphocreatine kinase activities (CK) were found to decrease under hypoxic conditions, indicating a reduced aerobic metabolism capacity [52]. To cope with hypoxia stress, certain regulatory enzymes involved in anaerobic and aerobic metabolisms may be downregulated or modified [53]. Gracey et al. found that in the euryoxic fish *Gillichthys mirabilis,* genes related to protein synthesis and movement were initially downregulated to reduce energy expenditure, while genes related to anaerobic ATP production and gluconeogenesis were upregulated to promote survival under hypoxic conditions. These processes inhibit cell growth and metabolic processes that heavily depend on energy, thus allowing the animal to enter a state of lower metabolism under hypoxia [54].

In this study, after 2 h of hypoxia stress, the Na^+^-K^+^-ATPase activity in the heart was significantly decreased, possibly due to enhanced anaerobic metabolism in the early stages of hypoxia stress, resulting in metabolic suppression. After 4 h of hypoxia stress, the heart COO activity began to increase, indicating an enhancement in aerobic metabolism that may have led to increased Na^+^-K^+^-ATPase activity. In the later stages of the experiment, the Na^+^-K^+^-ATPase activity in the liver and gills was lower in the HG than in the CG or not significantly different between the two groups. This result may be indicative of a different metabolic response in the heart when compared with the liver and gills. However, limited research has focused on hypoxia responses in tilapia hearts. Further investigations, including histology, transcriptomics, and metabolomics studies, are required to explore the molecular mechanisms underlying hypoxia-induced stress responses in tilapia hearts.

### 4.3. Hypoxia-Induced Morphological Adaptations in the Gills and Livers of Male GIFT

Fish gills are in direct contact with water, making them more susceptible to damage under hypoxia stress [49]. Previous studies have shown that exposing fish to hypoxic environments leads to structural changes in the gills within hours to days [2,4]. In the present study, the lamellae in gill filaments gradually swelled and became hyperemic after 8–24 h of hypoxia stress. A similar phenomenon has been reported in Atlantic stingrays (*Dasyatis sabina*) [55] and goldfish [56]. Congestion within gill filaments may facilitate increased gas exchange and oxygen absorption. Gill lamella elongation was observed in Lake Qinghai scaleless carp, suggesting that the gill filament structure may undergo adaptive changes under hypoxic stress [5]. In our study, with prolonged exposure to hypoxic conditions, the gill filament length gradually increased, while the interstitial cell cluster thickness decreased. These changes favor an increase in the respiratory surface area. These changes, as well as the changes in antioxidant enzyme activity, demonstrate that the gill tissues of male GIFT are able to adapt to a hypoxic environment via structural modifications.

The liver is a crucial metabolic organ in fish, and it is particularly sensitive to changes in DO levels in water. A previous study showed that the liver cells in golden pompano (*Trachinotus ovatus*) become vacuolated, displaying disordered (or, in severe cases, localized) hepatocyte necrosis under hypoxia stress [57]. Similar changes occurred in high-latitude fish (*Phoxinus lagowskii*) under hypoxia stress [58]. In the present study, we observed hepatic cell vacuolation after 8 to 24 h of hypoxia stress. Concurrently, the MDA content increased in liver tissue. We speculate that excessive amounts of ROS may have caused oxidative stress damage to liver cells, potentially resulting in vacuole formation. After 72 h of hypoxic stress, the hepatic MDA concentration was reduced to nearly normal levels and liver vacuolation was partially ameliorated. This may be because the DO level of 1.0 mg/L did not exceed the tolerance threshold of male GIFT, and the liver was able to adapt to the hypoxic conditions as a result of physiological and metabolic changes.

## 5. Conclusions

In this study, we conducted a comprehensive analysis of the antioxidant and metabolic responses of the gills, liver, and heart of male GIFT to acute hypoxia stress. Combined with histological analyses, our research revealed that hypoxia-induced oxidative stress occurred earlier in the heart and gills of male GIFT than in the liver. The hypoxia-stressed male GIFT exhibited an increased reliance on anaerobic metabolism as an adaptive response, effectively alleviating the metabolic burden induced by hypoxia. These insights shed light on the complex mechanisms through which male GIFT respond to hypoxia, contributing to our understanding of the physiological responses of fish to low-DO environments.

## Figures and Tables

**Table 1:** Changes in liver histological parameters of male GIFT under hypoxia stress.

Histological Parameter	Stress Time			
	0 h	8 h	24 h	72 h
Relative vacuole area (%)	6.05 ± 0.81 ^a^	18.57 ± 2.07 ^b^	37.59 ± 2.30 ^c^	27.47 ± 3.42 ^d^

Note: Relative vacuole area (%) = (vacuole area/total liver area) × 100. Different letters indicate statistically significant differences (*p* < 0.05) among different time points under hypoxia stress; *n* = 6.

**Table 2:** Changes in gill histological parameters of male GIFT under hypoxia stress.

Histological Parameter	Stress Time			
	0 h	8 h	24 h	72 h
Length of gill lamellae (μm)	70.26 ± 1.46 ^a^	78.01 ± 1.24 ^b^	81.44 ± 2.49 ^b^	97.60 ± 3.88 ^c^
Thickness of ILCM (μm)	26.55 ± 0.94 ^a^	22.13 ± 0.59 ^b^	12.90 ± 0.92 ^c^	10.15 ± 0.28 ^d^

Note: ILCM, interlamellar cell mass. Different letters indicate statistically significant differences (*p* < 0.05) among different time points under hypoxia stress; *n* = 6.

## Data Availability

All of the data are included in the article.
